# Polymerization Dynamics of the Prophage-Encoded Actin-Like Protein AlpC Is Influenced by the DNA-Binding Adapter AlpA

**DOI:** 10.3389/fmicb.2017.01429

**Published:** 2017-08-02

**Authors:** Aaron J. Forde, Nadine Albrecht, Andreas Klingl, Catriona Donovan, Marc Bramkamp

**Affiliations:** Fakultät für Biologie, Ludwig-Maximilians-Universität München Munich, Germany

**Keywords:** AlpC, AlpA, *alpS*, actin, phage, *Corynebacterium*, replication, DNA segregation

## Abstract

The *Corynebacterium glutamicum* ATCC 13032 prophage CGP3 encodes an actin-like protein, AlpC that was shown to be involved in viral DNA transport and efficient viral DNA replication. AlpC binds to an adapter, AlpA that in turn binds to specific DNA sequences, termed *alpS* sites. Thus, the AlpAC system is similar to the known plasmid segregation system ParMRS. So far it is unclear how the AlpACS system mediates DNA transport and, whether AlpA and AlpC functionally interact. We show here that AlpA modulates AlpC filamentation dynamics in a dual way. Unbound AlpA stimulates AlpC filament disassembly, while AlpA bound to *alpS* sites allows for AlpC filament formation. Based on these results we propose a simple search and capture model that explains DNA segregation by viral AlpACS DNA segregation system.

## Introduction

Maintenance and stable inheritance of genetic material is a fundamental problem in all living cells. Beside the bulk genetic information on the chromosome, bacteria often contain self-replicable DNA molecules, termed plasmids. Stable maintenance of low copy number plasmids is ensured by active segregation systems (Cabeen and Jacobs-Wagner, 2010; Gerdes et al., 2010; Salje et al., 2010; Schumacher, 2012). Plasmid segregation systems are classed as type I–III. Type I (ParA-like) are defined by ParA proteins characterized by a deviant walker A motif, type II relies on actin-like proteins ALPs (ParM like) and type III are tubulin (TubZ) based (Gerdes et al., 2010; Salje et al., 2010). Common to these partitioning systems is that they function as simple tripartite structures. A DNA sequence serves as centromere-like structure to which an adapter protein binds. The loaded adapter in turn binds to a nucleotide hydrolyzing protein that either serves as a molecular gradient or polymer giving direction to the DNA segregation process (Gerdes et al., 2010; Salje et al., 2010; Schumacher, 2012).

The actin-like ParM and AlfA systems are particularly well studied examples of plasmid encoded type II systems (Jensen and Gerdes, 1997, 1999; van den Ent et al., 2002; Moller-Jensen et al., 2003; Polka et al., 2009, 2014; Gayathri et al., 2012, 2013; Bharat et al., 2015). The ParMRC system consists of the actin-like ParM motor protein, the adapter protein ParR, and a centromeric region termed *parC* (Dam and Gerdes, 1994; Jensen and Gerdes, 1997, 1999; Gerdes et al., 2010). The *parMRC* locus was originally isolated from the multiple-antibiotic resistant R1 plasmid of *E. coli* (Dam and Gerdes, 1994; Jensen and Gerdes, 1997). In the presence of ATP, ParM forms filaments that either undergo catastrophic disassembly upon nucleotide hydrolysis or are stabilized by becoming capped with a ParR–*parC* complex (Jensen and Gerdes, 1997, 1999; Garner et al., 2007; Gayathri et al., 2012; Bharat et al., 2015). Once stabilized, the filament elongates in both directions thereby pushing plasmids in opposite directions (Garner et al., 2007). The instability of uncapped ParM leads to cycles of growth and disassembly and it is in this way that ParM filaments search for plasmids within the cell space (Garner et al., 2004, 2007). ParM assembles into polar, twisted double filaments that wrap around each other in a left handed arrangement (van den Ent et al., 2002; Popp et al., 2008; Galkin et al., 2009). The pBET131 plasmid of *Bacillus subtilis* uses AlfA filaments for segregation during growth and sporulation (Becker et al., 2006). Similar to ParM, the AlfA system consists of a motor protein (AlfA), an adapter protein (AlfB) and a centromeric DNA sequence *parN.* AlfA and ParM share only 15% sequence similarity (Becker et al., 2006; Derman et al., 2012). In the presence of either ATP or GTP, AlfA forms twisted, helical filaments with a left handed turn (Becker et al., 2006) with addition of subunits occurring only at one end of the filament. AlfA shows self-assembly properties and forms large bundles of mixed polarity. AlfA filaments are dynamic and utilize treadmilling of so called comet tails to push plasmids (Polka et al., 2009, 2014). Treadmilling is a process in which one end of the filament grows while proteins from the other end are released. At steady state this growth and loss are in equilibrium. Usually proteins that undergo treadmilling need a critical concentration of subunits. In contrast to ParM, AlfA filaments have not been observed to undergo catastrophic disassembly. Instead, AlfA assembly and disassembly is regulated by the adapter protein AlfB (Polka et al., 2014). Free AlfB breaks up AlfA bundles, but when AlfB is bound to *parN*, it forms a segrosome complex that stabilizes AlfA filaments. Two mechanisms are proposed as to how AlfB destabilizes AlfA bundles. Free AlfB is thought to prevent bundle formation either by binding to the sides of AlfA filaments and preventing their lateral association or by decreasing the lifetime of AlfA filaments so they do not have time to from bundles. It is also suggested that AlfB might cap AlfA filaments or sequester AlfA monomers (Polka et al., 2014).

Actin-like proteins (ALPs) are ubiquitously distributed in all kingdoms of life. A recent phylogenetic analysis, led to the identification of more than 35 families of ALPs in bacteria (Derman et al., 2009). While some of these were found on bacterial chromosomes, most were located on phage genomes, plasmids, or integrating conjugative elements (Derman et al., 2009). ALPs are involved in a variety of cellular functions that range from spatial coordination of cell wall synthesis proteins, subcellular positioning of protein complexes and magnetosomes to segregation of DNA (Cabeen and Jacobs-Wagner, 2010; Cowles and Gitai, 2010; Errington, 2015; Toro-Nahuelpan et al., 2016).

We have recently described the CGP3 prophage in the *Corynebacterium glutamicum* genome (Frunzke et al., 2008; Donovan et al., 2015). Examination of the CGP3 genomic region revealed that the first open reading frame encodes an actin-like protein, which has been designated AlpC (*ac*tin-like *p*rotein *C*orynebacterium; Donovan et al., 2015). CGP3 has been shown to be activated by DNA-damaging agents such as mitomycin C, however, a low percentage of cells in a growing *C. glutamicum* culture have induced and replicating prophage (Frunzke et al., 2008; Nanda et al., 2014, 2015; Donovan et al., 2015; Helfrich et al., 2015).

While AlpC shares low sequence identity with actin and other actin-like proteins it does contain the typical actin signature motif (Donovan et al., 2015). AlpC has been shown to hydrolyze nucleotides and form dynamic, filamentous structures, both of which are characteristic features of actin and actin-like proteins. When expressed at native levels, AlpC assembled into short, straight filaments, in contrast, when overexpressed it assembles into long curved filaments. The co-transcribed adapter protein AlpA has been shown to interact with AlpC. In addition, AlpA binds to a DNA consensus sequence, termed *alpS*, in the upstream promoter region of the *alpAC* operon (Donovan et al., 2015). Thus, in a proposed model, AlpA binds to *alpS* as well as interacting with AlpC, in this way circular phage DNA is connected to the actin-like filament. Evidence that both proteins are important for efficient phage replication was provided by transcriptome analyses that revealed *alpC* and *alpA* are among the first genes to be induced following exposure to Mitomycin C. Additionally, mutants lacking either AlpA or AlpC had phage DNA levels that were approximately twofold reduced when compared to the wild type (Donovan et al., 2015). Based on these observations, we hypothesized that CGP3 may utilize AlpC filaments as a transport mechanism through the cell space in order to reach a preferred site of replication (Donovan et al., 2015). Unlike other rod-shaped bacteria, *C. glutamicum* lacks other actin-like cytoskeletal proteins (Donovan and Bramkamp, 2014), it may therefore be advantageous for CGP3 to encode its own cytomotive element for transport within the cell space.

Up to now, little is known about the molecular mechanisms of how phage encoded ALPs catalyze DNA segregation. We have therefore analyzed the AlpACS system *in vitro* using purified components. Using ATP hydrolysis assays, dynamic light scattering (DLS) and negative stain electron microscopy we were able to show that AlpA influences AlpC filament formation in an *alpS* dependent manner. While unbound AlpA leads to AlpC filament disassembly, *alpS*-loaded AlpA stabilizes AlpC filaments. The data are in good agreement with a simple search and capture mechanism, by which AlpAC drive viral DNA segregation.

## Materials and Methods

### Bacterial Strains, Plasmids, and Oligonucleotides

Bacterial strains, plasmids, and oligonucleotides used in this study are listed in Supplementary Tables S1–S3.

### Media and Antibiotics

Bacteria were grown in Lysogeny broth with appropriate antibiotic concentrations (carbenicillin, 100 μg ml^-1^; chloramphenicol, 30 μg ml^-1^; kanamycin, 25 μg ml^-1^).

### General Molecular Biology Techniques

Molecular cloning was carried out using standard techniques (Sambrock et al., 1989). Plasmids were maintained in *E. coli* DH5α cells (Invitrogen).

### Heterologous Protein Overexpression

For the overexpression of pET16B-GFP-AlpC and pET16B-mCherry-AlpA, the Lemo21(DE3) strain was used. Plasmid DNA was transformed followed by overnight incubation, cells were inoculated in a starter culture and grown to an optical density (OD_600_) of approximately 1. Next, 700 ml main cultures were inoculated to an OD_600_ of 0.1 and incubated at 37°C with shacking at 200 rpm. Once an OD_600_ of 0.6 was reached, the main cultures were induced by addition of 400 μM IPTG and 200 μM L-rhamnose and grown overnight at 19°C with shaking at 160 rpm. Overexpression of pETTEV-AlpA and pET16b-AlpC were performed in the same manner as described above with the exception that the cultures were induced with 400 μM IPTG only. After overnight incubation cultures were harvested by centrifugation at 8000 *g* for 30 min at 4°C. The supernatant was removed and the cell pellets were flash frozen in liquid nitrogen and stored at -80°C.

### Affinity Chromatography

Frozen pellets containing the protein of interest were thawed on ice and resuspended in binding buffer (50 mM Tris/HCl pH 7.5, 150 mM NaCl, 150 mM KCl, 10% glycerol, 10 mM imidazole) supplemented with DNase I and protease inhibitor. The resuspended cultures were lysed with a French Press and centrifuged at 8000 *g* for 30 min to remove cell debris. The supernatant was then spun in an ultracentrifuge at 200,000 *g* for 45 min to remove membranes. The cleared cell lysate was applied to an Äkta FPLC and run over a Ni-NTA agarose column (1 ml Protino-Ni-NTA column, Macherey and Nagel, Düren, Germany). Elution was done in elution buffer (50 mM Tris/HCl pH 7.5, 150 mM NaCl, 150 mM KCl, 10% glycerol, 500 mM imidazole) and fractionated in volumes of 1 ml. Following elution, samples from each fraction were analyzed by SDS-polyacrylamide gel electrophoresis (SDS-PAGE).

### Size Exclusion Chromatography

Affinity purified protein was applied to size exclusion chromatography (SEC) performed on an Äkta FPLC system (GE Healthcare). The proteins were separated using either the Superdex 200 or Superose 6 columns (GE Healthcare). SEC was performed in buffer containing 50 mM Tris/HCl, pH 7.5, 25 mM NaCl, 25 mM KCl, and 10% glycerol. The flow rate was set to 0.4–0.5 ml/min. Fractions were collected in 500 μl volume and analyzed by SDS-PAGE. Purified protein was concentrated in centrifugal filter devices with 10 kDa size exclusion (Millipore) by centrifugation. Samples were mixed several times during the process. The protein concentration was measured by a bicinchoninic acid assay (Thermo Fisher Scientific) according to the manufactures instructions.

### SDS-Polyacrylamide Gel Electrophoresis

Purified protein fractions were examined using SDS-PAGE. A 10% gel resolving gel with 4% stacking gel was used. Samples were supplemented with 4% SDS loading buffer (200 mM Tris/HCl pH 6.8, 400 mM DTT, 8 % SDS, 0.4% bromophenol blue, 40% glycerol) and were incubated for 15 min at 39°C before loading on the gel. Gels were run in a Bio-Rad chamber in SDS Laemmli buffer (25 mM Tris, 192 mM glycine, 0.1% SDS) at 120–150 V. After the electrophoresis run, gels were stained overnight in Coomassie blue solution. Gels were de-stained in 10% acetic acid or in destaining solution (H_2_O, methanol, and acetic acid mixed in a 50/40/10 ratio).

### Immunoblotting

Proteins were transferred to a polyvinylidene difluoride membrane using the wet blot method in transfer buffer (132 mM glycine, 25 mM Tris) containing 20% methanol. Transfer was performed either at 75 mA overnight or at 200 mA for 2 h. Following the transfer, the membranes were blocked in a 5% milk solution (5% milk powder in wash buffer) overnight at 4°C. The membrane was then washed three times for 5 min in wash buffer (50 mM Tris/HCl pH 7.5, 150 mM NaCl) before application of the first antibody. The first antibody was incubated for 1 h at room temperature, succeeded by another round of washing. Next, the appropriate secondary antibody was applied and again incubated for 1 h at RT. A final round of washing steps was performed before the membrane was developed in incubation buffer (100 mM Tris, 100 mM NaCl, 5 mM MgCl_2_, 6× H_2_0, and 0.02% NaN_3_) supplemented with NBT and BCIP.

### Primer Pair Annealing

Equal molar amounts of each primer were mixed and heated at 95°C for 5 min. After the incubation period, the heat block was switched off while the samples remained in place and allowed to gradually cool to room temperature. Annealed primers were stored at -20°C.

### Electrophoretic Mobility Shift Assays

To determine if mCherry-AlpA binds to the annealed *alpS* DNA with a Cy5 modification an electrophoretic mobility shift assay (EMSA) was performed. Increasing molar concentrations of mCherry-AlpA were incubated with 500 ng of *alpS* DNA for 30 min at 30°C. Samples were supplemented with 2× clear native buffer and loaded onto a 10% non-denaturing PAGE and run at 4°C at 150 V. Samples were visualized using a Typhoon scanner (GE Healthcare).

### Sedimentation Assay

To determine if AlpC formed oligomeric structures in the presence of ATP a sedimentation assay was performed. Three micromolar AlpC was incubated with 1 mM ATP and 1 mM MgCl_2_.

EDTA was used to abolish sedimentation in control experiments. Samples were incubated for 30 min at 30°C and then spun down at 28,000 *g* for 1 h after which the supernatant was removed, the pellet was resuspended in an equal volume of SEC buffer. The samples were mixed in 4× SDS loading buffer and then run on a 10% SDS-PAGE and stained with Coomassie blue.

### Dynamic Light Scattering

DLS was used to study AlpC polymerization. Appropriate amounts of protein, ATP, MgCl_2_, and buffer were incubated in a 100 μl cuvette at 30°C for 30 min. The sample was measured using the DynaPro NanoStar (Wyatt Technology), the system was set to 30°C and recorded an acquisition every 5 s for a total of 50 acquisitions. Prior to beginning an experiment, SEC buffer and MgCl_2_ were passed through a 2 μM filter. Next, all components were centrifuged at 200,000 *g* for 10 min in order to pellet any aggregates or other debris that might interfere with the measurements.

### ATP Hydrolysis Assay

Purified AlpC protein (1 μM) was incubated with increasing concentrations of ATP (50–1000 μM), 1 mM MgCl_2_, and SEC buffer in a total volume of 150 μl. The samples were incubated for 30 min at 30°C, after incubation the samples were passed through a 0.2 μM filter. Samples analyzed by high-performance liquid chromatography (HPLC) in 20 μl volumes and passed through a reverse phase C18 column in ATPase assay buffer (10 mM tetra-*n*-butylammonium bromide, 100 mM K_2_HPO_4_, 100 mM KH_2_PO_4_, 0.2 mM NaN_3_, and 2% acetonitrile). The buffer has a pH of 6.5, the acetonitrile was added last after the buffer containing the other components was passed through a 2 μM sterile filter. Each sample was measured in triplicate and the average measurement of the peak areas was obtained.

### Electron Microscopy

Negative staining of purified samples was carried out with 2% uranyl acetate as described before (Zander et al., 2017). Transmission electron microscopic analysis was carried out using a Zeiss EM 912 with an integrated OMEGA energy filter, which was operated at 80 kV in the zero-loss mode. Image acquisition and analysis was performed using the Tröndle imaging software (TRS Tröndle Restlichverstärkersysteme, Moorenweis, Germany).

## Results

### Purification of AlpA and AlpC

For characterization of the biochemical properties of the prophage encoded actin-like protein AlpC and its adapter protein AlpA the corresponding genes were cloned into expression vectors to allow for overexpression in *E. coli* as described in the Section “Materials and Methods.” AlpA and AlpC were produced as fluorescent fusions proteins (GFP-AlpC and mCherry-AlpA) and with a histidine-tag to allow for metal-affinity chromatography. The reasoning behind the use of fluorescent fusion proteins was that we wanted to compare *in vitro* AlpC polymer formation that was observed *in vivo* using fluorescent fusion proteins (Donovan et al., 2015). As control, we also purified and analyzed the native proteins without fluorescent fusion. Additionally a catalytically inactive AlpC mutant, AlpC-D301A, was purified. Protein overexpression was performed in Lemo21(DE3) cells. Overexpression of proteins using T7-based transcription systems often results in generation of insoluble protein aggregates due to massive mRNA levels. Therefore, we used the tunable expression system of the Lemo21(DE3) strain. Expression in these cells is tuned by addition of various L-rhamnose concentrations to the expression culture. Rhamnose modulates the level of lysozyme (encoded by *lysY*) that acts as inhibitor of the T7 RNA polymerase. Protein synthesis was carried out at 19°C over night to further reduce inclusion body formation. Clear cell lysates were subjected to metal affinity chromatography using Ni-NTA columns on an Äkta FPLC system. Elution of the recombinant proteins was achieved by a step gradient with increasing imidazole concentrations. GFP-AlpC and mCherry-AlpA eluted in concentrated fractions with some smaller degradation products (Supplementary Figure S1A). Pooled elution fractions containing AlpC or AlpA proteins were subjected to SEC on a Superdex 200 or Superose 6 column (Supplementary Figure S1B). Degradation products of GFP-AlpC and mCherry-AlpA could be separated well and peak fractions with concentrated full-length proteins were pooled and used for further analysis.

### AlpA Influences ATP Hydrolysis of AlpC

The hydrolysis of nucleotides is one of the hallmarks of ALPs. We have shown before that AlpC hydrolyzes ATP and links its hydrolysis cycle to polymerization (Donovan et al., 2015). We now wanted to address whether ATPase activity of AlpC was influenced by the accessory protein AlpA.

**FIGURE 1:**
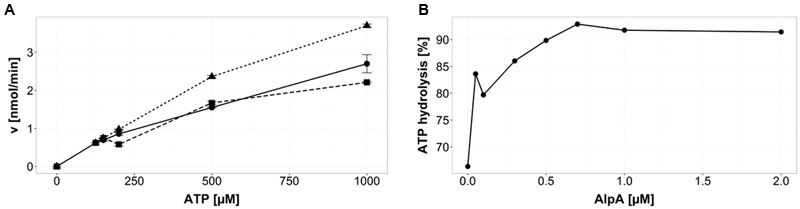
AlpA influences AlpC nucleotide hydrolysis. **(A)** ATP hydrolysis of AlpC (1 μM) is increased by free AlpA. ATP hydrolysis of GFP-AlpC (solid line with filled spheres) is shown with increasing ATP concentrations. Addition of 1 μM mCherry-AlpA accelerates ATP hydrolysis of AlpC (dotted line with filled triangles). Addition of AlpA that was pre-incubated with *alpS* DNA reduces ATP turnover (broken line with filled squares. Error bars are showing SEM. **(B)** Equimolar concentrations of AlpA accelerate AlpC ATP hydrolysis. ATP hydrolysis of 1 μM GFP-AlpC was measured. Addition of increasing concentrations of mCherry-AlpA stimulates nucleotide turnover until saturation is reached at about equimolar concentrations of AlpC and AlpA. Note that GFP-AlpC has about 60% of its maximal activity in absence of AlpA.

ATP hydrolysis was analyzed using HPLC analysis. By comparing the peak areas of the samples to the peak areas of ATP standards the remaining concentration of ATP present could be calculated. From this, the rate of hydrolyzed ATP at each concentration was determined. One micromolar purified GFP-AlpC was incubated with increasing ATP concentrations ranging from 50 to 1000 μM and incubated at 30°C for 30 min. The rate of hydrolysis increases and has not reached a saturation at 1000 μM ATP (**Figure 1A**, solid line). Next, it was tested if mCherry-AlpA may affect ATP hydrolysis in AlpC. Therefore, 1 μM GFP-AlpC was incubated together with 1 μM mCherry-AlpA at the same ATP concentrations and incubation times as taken for GFP-AlpC alone. In these samples the rate of ATP hydrolysis was markedly increased (**Figure 1A**, dotted line). We conclude that presence of AlpA triggers ATP hydrolysis in AlpC. Finally, a third set of samples consisting of 1 μM GFP-AlpC, 1 μM mCherry-AlpA, and 1 μM *alpS* DNA was examined. The mCherry-AlpA and *alpS* DNA were allowed to bind before being added to the rest of the components, formation of the AlpA–*alpS* nucleoprotein-complex was facilitated by incubation for 30 min at 30°C. After addition of preloaded AlpA the rate of ATP hydrolysis returned to levels similar to AlpC alone or even below (**Figure 1A**, broken line). AlpA did not reveal any nucleotide hydrolysis activity (not shown). These data point to the fact that AlpA bound to *alpS* does not stimulate ATP hydrolysis in AlpC, but in fact may rather slow it down slightly. AlpC displays basal ATPase activity that is increased by addition of AlpA. Titration of AlpA reveals that approximately equimolar AlpA and AlpC show highest ATP hydrolysis rates (**Figure 1B**).

### AlpC Forms Higher Oligomeric Structures *In Vitro*

**FIGURE 2:**
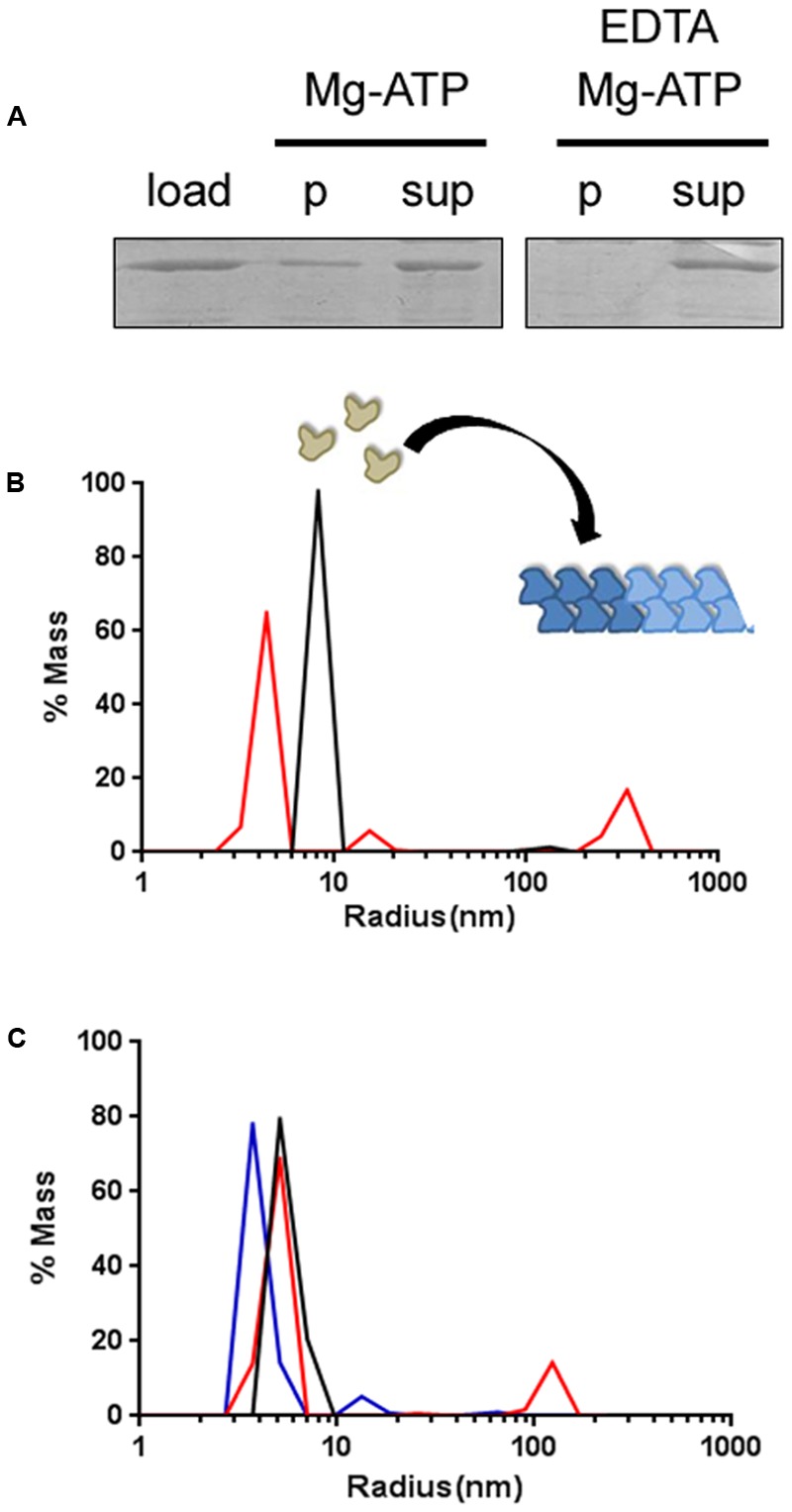
GFP-AlpC forms large polymeric assemblies following ATP incubation. **(A)** Sedimentation assay reveals the polymerization of GFP-AlpC after ATP incubation. One micromolar AlpC was incubated with Mg^2+^ and ATP (1 mM). After centrifugation a substantial part of AlpC sediments due to polymer formation. Polymerization is Mg-ATP dependent, since addition of EDTA prohibits AlpC sedimentation (p, pellet fraction; sup, supernatant; load, total protein). **(B)** Using dynamic light scattering, ATP induced GFP-AlpC filament formation was observed. In absence of ATP a single protein species is detected indicating primarily monomeric/dimeric species (black line). Incubation with ATP results in the detection of peaks with increased radii demonstrating the formation of a larger protein species (red line). **(C)** Addition of free mCherry-AlpA in equimolar concentrations to AlpC results in GFP-AlpC de-polymerization (blue line), giving similar hydrodynamic radii as found when GFP-AlpC was incubated without ATP (black line). Polymerization of GFP-AlpC in presence of ATP is depicted by a red line.

First, we revisited earlier results of AlpC polymerization using sedimentation assays. This, was to prove that the GFP-AlpC fusion protein would behave similar to the untagged version. Addition of Mg-ATP lead to polymerization of GFP-AlpC (**Figure 2A**). In control experiments, we added EDTA to chelate magnesium. Under these conditions no GFP-AlpC was detected in the sediment (**Figure 2A**). Thus, the GFP-AlpC fusion protein behaved comparable to the native AlpC protein that was analyzed before (Donovan et al., 2015). We next wanted to analyze the oligomerization behavior of AlpC using DLS. With this analysis the hydrodynamic radius of a given protein sample can be analyzed. Therefore, 1 μM AlpC was mixed with SEC buffer including 200 μM MgCl_2_ and measured subsequently. Each measurement consists of 50 acquisitions with 5 s interval time. In the absence of ATP AlpC did not polymerize into larger oligomers (**Figure 2B**), confirming earlier observations with sedimentation assays (Donovan et al., 2015). This species has an estimated molecular weight of around 58 kDa, given that GFP-AlpC has a molecular weight of 77.5 kDa this species probably represents monomers. Next, 200 μM ATP were added and a new measurement taken to see if a larger protein species was detected after incubation at 30°C for 30 min. Upon incubation with ATP two new peaks were detected. The peak associated with the largest radius has an estimated molecular weight of 256,600 kDa, indicating that AlpC has polymerized after incubation with ATP and formed large structures (**Figure 2B**).

Next, we wanted to address whether addition of N-terminal fusion of a fluorescent protein might interfere with the polymerization of AlpC *in vitro*. Purified AlpC without a fluorescent fusion was therefore introduced to DLS measurements using identical settings as used for the tagged AlpC. Again, addition of ATP triggered AlpC polymerization in a comparable way as observed for the fusion protein, indicating that addition of an N-terminal GFP fusion does not abolish the ability of AlpC to form higher order structures (Supplementary Figure S2A). As control we used the catalytically inactive AlpC-D301A mutant. The purified AlpC-D301A protein forms no oligomers in the presence of ATP, confirming that the increase in particle size observed for the wild-type protein is clearly ATP-dependent (Supplementary Figure S2B). Similar to AlfA, AlpC forms filamentous structures with ADP (Supplementary Figure S2C).

### AlpA Inhibits Formation of AlpC Oligomeric Structures

For the plasmid segregation protein AlfA it has been shown that the adapter protein AlfB influences AlfA polymer dynamics (Polka et al., 2014). Similarly, we wanted to test whether the adapter protein AlpA influences AlpC polymerization. Incubation of GFP-AlpC with ATP resulted in the formation of oligomers as expected. However, when mCherry-AlpA and GFP-AlpC were incubated together in the presence of ATP the large molecular assemblies were absent, suggesting that AlpA leads to disassembly of AlpC polymers or blocked AlpC polymerization (**Figure 2C**). Again, we tested whether the native proteins react in a similar manner. We mixed AlpC with AlpA and again no large oligomers of AlpC were apparent when the mixture was incubated with ATP. We conclude from this experiment that an N-terminally tagged AlpA still retains the ability to inhibit AlpC polymerization (Supplementary Figure S2A).

### AlpA Bound to *AlpS* Stabilizes AlpC Filaments

**FIGURE 3:**
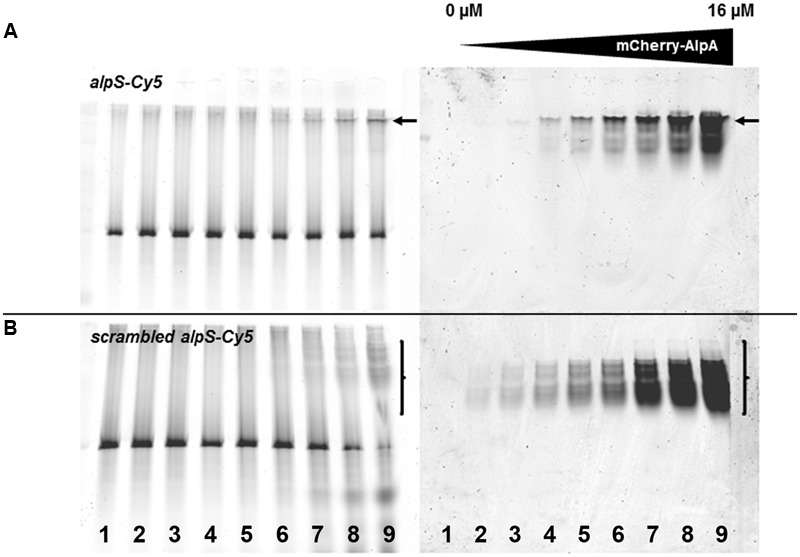
mCherry-AlpA binds to *alpS* DNA *in vitro*. EMSA with purified mCherry-AlpA binding *alpS*-Cy5 consensus sequence **(A)** and a scrambled *alpS* sequence **(B)**. Increasing concentrations of mCherry-AlpA were used [lane 1: 0 μM (DNA only), lane 2: 0.125 μM, lane 3: 0.25 μM, lane 4: 0.5 μM, lane 5: 1 μM, lane 6: 2 μM, lane 7: 4 μM, lane 8: 8 μM, lane 9: 16 μM) to shift 500 ng double stranded DNA. Loading was identical for **(A)** and **(B)**. Note the different behavior of binding. mCherry-AlpA shifts *alpS*-Cy5 to a sharp, highly defined band (arrow), while binding to scrambled *alpS* sequences results in a smear of lower shifted bands (bracket).

AlpA binds to a DNA sequence termed *alpS* (TTAAnnG), which is repetitively found in the *alpCA* promoter region (Donovan et al., 2015). We therefore wanted to find out whether AlpA bound to its target sequence *alpS* would differently interfere with AlpC polymerization. An *alpS* containing DNA was constructed by annealing complementary primer pairs that encompass seven *alpS* repeats. Binding of mCherry-AlpA to the double stranded *alpS* DNA was tested by EMSA (see Materials and Methods). For this, increasing concentrations of mCherry-AlpA (0–16 μM) were incubated with 500 ng *alpS* DNA labeled with Cy5 dye. Binding of AlpA to its DNA target is shown by shifting the DNA sequence (**Figure 3**). Specificity of this binding was tested by offering a scrambled *alpS* sequence in which the bases have been shuffled. Although some residual, unspecific binding of AlpA was observed, the binding was reduced (**Figure 3**). Strikingly, the nature of the shifted bands was different. AlpA-binding to *alpS* DNA resulted in a sharp, highly shifted band that also accumulated the majority of the mCherry-AlpA (**Figure 3A**, arrows). In contrast, binding of mCherry-AlpA to scrambled *alpS* resulted in a series of lower shifted bands and a smear of mCherry-AlpA (**Figure 3B**, bracket). Apparently, the correct *alpS* sequence induces a different oligomerization of AlpA. However, AlpA is still able to bind to unspecific (scrambled) DNA to some extent.

**FIGURE 4:**
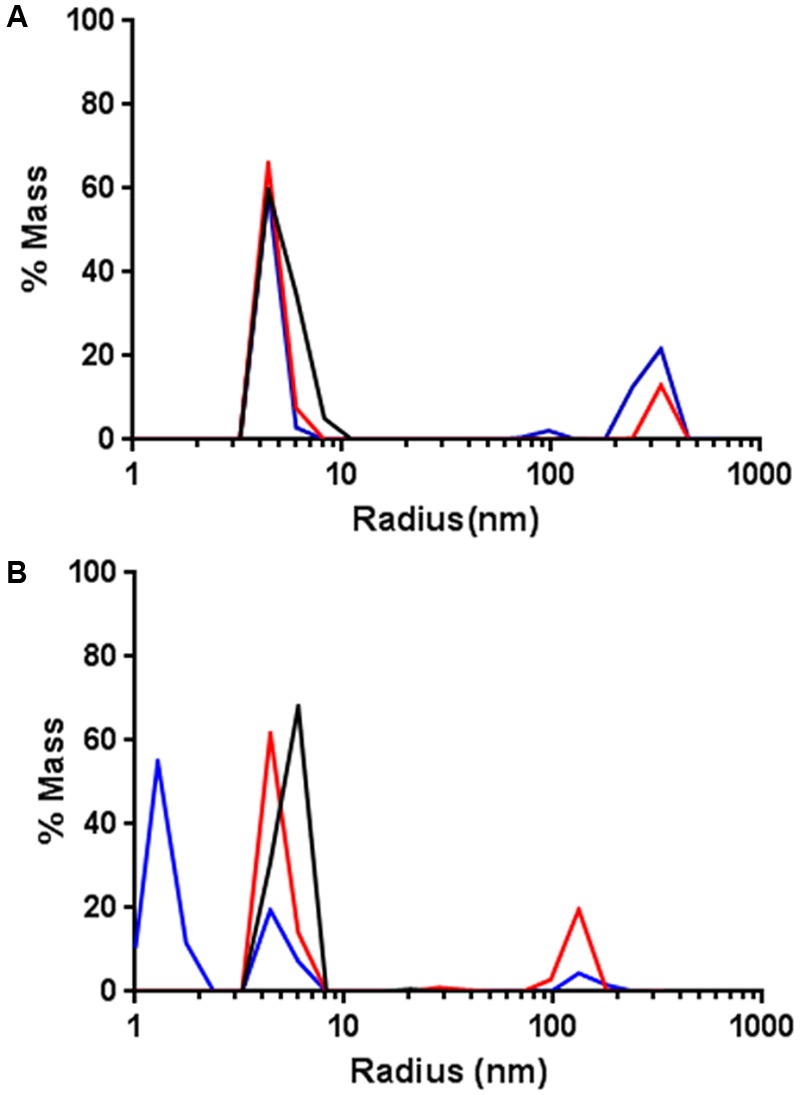
AlpA–*alpS* nucleoprotein complexes stabilize AlpC filament formation. **(A)** Addition of pre-bound mCherry–AlpA–*alpS* complexes does not result in GFP-AlpC de-polymerization, but rather stabilizes the GFP-AlpC polymer fraction. Black lines indicate GFP-AlpC without ATP, red lines show GFP-AlpC with ATP, and blue lines indicate polymerization of GFP-AlpC in presence of mCherry–AlpA–*alpS* complexes. **(B)** Addition of a pre-bound scrambled mCherry-AlpA-scrambled-*alpS* mixtures results in depolymerization of GFP-AlpC polymers. Color code is as in **(A)** with blue line showing the effect of mCherry-AlpA with scrambled-*alpS.*

Based on these observations, the effect of DNA-bound AlpA was tested by adding the consensus sequence, *alpS* to the DLS measurement. In the DLS experiments, *alpS* DNA was used without the Cy5 modification. One micromolar GFP-AlpC was incubating with 200 μM ATP, 10 μM of *alpS* DNA was incubated with 1 μM mCherry-AlpA for 30 min at 30°C. Following this incubation, the *alpS*/mCherry-AlpA mixture was added to the GFP-AlpC and measured. In contrast to AlpA addition alone, no depolymerization effect was observed when AlpA–*alpS* nucleoprotein complexes were added (**Figure 4A**). Thus *alpS*-bound mCherry-AlpA does not stimulate GFP-AlpC depolymerization, but has rather an opposite effect in stabilizing the AlpC filament. Influence of *alpS* DNA was also tested with AlpC and AlpA lacking the fluorescent fusions. The experiment was carried out the same way as above with the result that addition of AlpA bound to *alpS* did not cause a depolymerization of AlpC and rather stabilized the polymer (Supplementary Figure S2D).

As a control experiment, addition of the scrambled *alpS* DNA sequences to AlpA was tested. Therefore, 10 μM scrambled *alpS* DNA were incubated with 1 μM mCherry-AlpA for 30 min at 30°C. As demonstrated by the EMSA experiments, these primers still bind to mCherry-AlpA, albeit with reduced affinity and a different mode of binding (**Figure 3**). Therefore, any potential stabilizing effect should be reduced with these primers. As in previous experiments, incubation of 1 μM AlpC with 200 μM ATP resulted in the detection of a high molecular weight species. Next, the bound AlpA/scrambled *alpS* complex was added to the cuvette. In contrast, to the correct *alpS* primers, the measurements revealed a drastic reduction in the mass of the high molecular weight species (**Figure 4B**).

**FIGURE 5:**
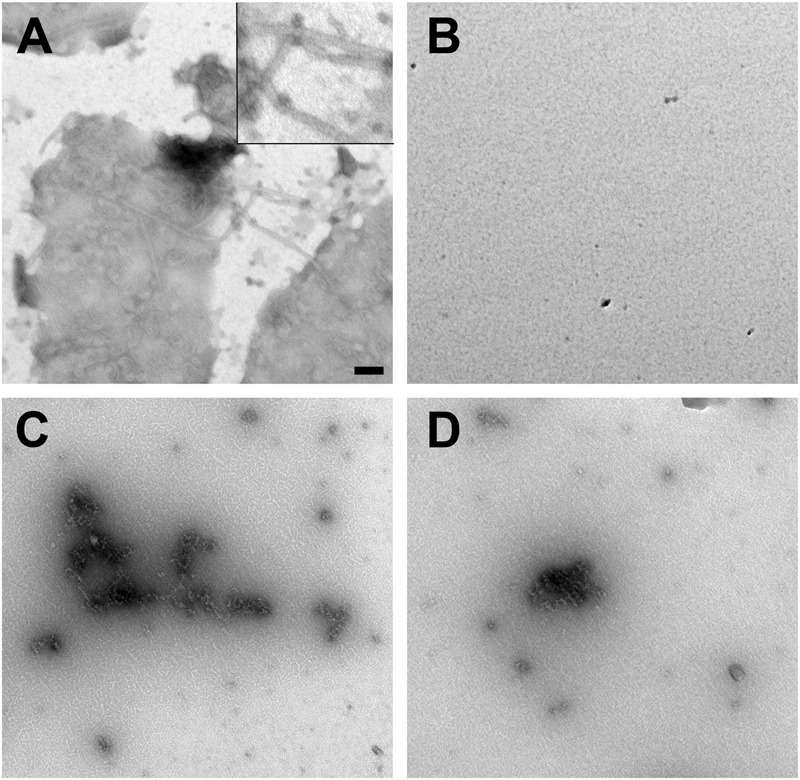
Negative stain electron microscopy. **(A)** GFP-AlpC (1 μM) forms straight filaments when incubated with ATP (1 mM). The inset in the top corner shows a magnification of the observed AlpC filaments. A statistical analysis of length and diameter is shown in Supplementary Figure S3. **(B)** Addition of mCherry-AlpA (1 μM) to the AlpC/ATP mix results in rapid loss of filaments. Preincubation of mCherry-AlpA with *alpS* sequences (10 μM) retains filament formation **(C)**, while preincubation with scrambled *alpS* triggers disassembly of AlpC filaments **(D)**. Images are taken at 16,000-fold magnification. Scale bar is 100 nm.

We corroborated the findings by negative stain election microscopy. Therefore, proteins were incubated similar to the light scattering experiments. AlpC was analyzed with and without ATP. Addition of ATP results in a clear filamentation of AlpC (**Figure 5A**). The observed filaments were straight and had an average length of 296.9 nm (SD 111.5 nm) and an average width of 19.4 nm (SD 2.6 nm) (Supplementary Figure S3). Addition of AlpA to the samples leads to disassembly of all visible filaments (**Figure 5B**). We than preincubated AlpA with *alpS* identical to the assay for the light scattering and added these preloaded AlpA–*alpS* complexes to the AlpC sample with ATP. Again, we were able to readily observe AlpC filaments. However, these filaments had a greater variety in length and diameter and had an overall less compact structure (**Figure 5C**). These AlpACS complexes had an average length of 230 nm (SD 147.7 nm) and an average diameter of 23.2 nm (SD 8.2 nm). We have summarized the statistical evaluation of the negative stain experiments in Supplementary Figure S3. We wanted to control for the specificity of the *alpS*-mediated effect and preincubated AlpA with scrambled *alpS* before addition to AlpC (in presence of ATP). As expected from the DLS results, the incubation with scrambled *alpS* lead to disassembly of the AlpC filaments almost similar to the situation with AlpA alone. Only occasionally short AlpC filaments were still observed (**Figure 5D**). Negative stain images of AlpA or AlpC alone did not reveal any structures (not shown). In summary, the electron microscopy images are in line with the data derived from light scattering experiments and support ATP-dependent filamentation of AlpC.

## Discussion

The *C. glutamicum* prophage CGP3 relies on a tripartite segregation system that has apparent homologies to type II segregation machineries of low copy number plasmids (Donovan et al., 2015). Here we report that AlpC filament dynamics are regulated by the adapter protein AlpA. AlpA is able to modulate AlpC filament dynamics in a dual way. When bound to its DNA-binding sequence *alpS*, the AlpA–*alpS* nucleoprotein complex stabilizes AlpC filament formation. This directly leads to increased filament growth once a cargo-loaded AlpA is encountered by an AlpC filament. In contrast, unloaded AlpA protein, in absence of *alpS*, leads to a rapid disassembly of AlpC filaments indicated by the loss of high molecular weight structures in our light-scattering experiments. An AlpC-D301A mutant is unable to form filamentous structures *in vitro*, similar to the corresponding mutations in Alp7A (D212A) (Derman et al., 2012). Mutation of the essential glutamate residue in ParM (D170A) and AlfA (D168D) results in loss of filament dynamics, but static filaments can still be observed (Jensen and Gerdes, 1997; Moller-Jensen et al., 2002; Becker et al., 2006). Thus, similar to other plasmid segregation systems such as the AlfAB system from *B. subtilis*, the filament formation is regulated in a dual way by its cognate adapter protein. It has been speculated that the AlfB dependent filament destabilization may have similarities to the cofilin interaction of eukaryotic actin (Polka et al., 2014). Cofilin helps to break down F-actin filaments, thereby enhancing rapid depolymerization and increasing the pool of G-actin (De La Cruz and Gardel, 2015). At present, we cannot rule out that AlpA is able to break up existing AlpC filaments by levering the filament structure. However, it seems more likely that AlpA interacts only with the barbed-end of the elongating AlpC filament as it has been shown for the ParR protein on ParM filaments. Data derived from negative stain also did not reveal any AlpC filaments of intermediary length after AlpA addition, suggestion that the depolymerization is fast and complete. The similar AlfA–AlfB interaction has also been shown to occur at the filament tips (Polka et al., 2014). There is good evidence that ParR–*parC* complexes stabilize growth at the barbed-end by a formin-like mechanism (Gayathri et al., 2012, 2013). Formin helps to bundle actin cables and filament bundling has been shown to be important for ParM-mediated plasmid segregation. We have shown earlier that AlpA foci seem to move on existing AlpC cables (Donovan et al., 2015). A likely explanation is that AlpA foci are pushed at the tip of an elongating AlpC filament that slides along other AlpC filaments. Therefore, AlpC is a clear example of cytomotive structures (Salje et al., 2010).

The AlpA protein has no homology to other adapter proteins such as ParR or AlfB. Therefore, it is currently unclear how it binds DNA and interferes with AlpC. Homology searches indicate that the N-terminal part of AlpA (residues 19–96) have weak homology to the AlkA N-terminal domain. The *E. coli* AlkA protein is involved in base excision repair and exhibits a helix-hairpin-helix (HhH) motif involved in DNA binding (Doherty et al., 1996; Bowman et al., 2008). DNA bound to AlkA is highly bent. Similarly, ParR-bound *parS* DNA was shown to be bent as well. However, ParR binds via a classical helix-turn-helix (HTH) motif to DNA (Schumacher et al., 2007). Thus, a common theme of adapter-DNA binding might be binding and deformation of DNA to facilitate interactions and segregation. The viral AlpACS segrosome is apparently no exception.

Interestingly, it seems that actin-like protein-based segregation systems fall in two differentially functional classes. ParM and Alp7A are dynamically unstable, shown by growing and shrinking filaments *in vivo* (Garner et al., 2004; Derman et al., 2009; Drew and Pogliano, 2011). Both proteins are supposed to undergo treadmilling. In contrast, AlfA and AlpC filaments do not show dynamic instability and likely do not treadmill (Polka et al., 2014; Donovan et al., 2015). In line with this notion is the fact that AlfA and AlpC form polymers in the presence of ADP (Polka et al., 2009) (Supplementary Figure S2C). These proteins seem to bundle filaments that may slide along each other. Indeed, recovery times determined in FRAP experiments are similar for AlfA and AlpC (Becker et al., 2006; Donovan et al., 2015). Although AlfA and ParM are both left-handed helices, they differ in their geometry, likely explaining the functional differences in their biochemistry. A detailed structure of the AlpC filaments still needs to be determined, but the similarities in the biochemical behavior might suggest similar filament architecture as in AlfA. A major difference between the ParMRC system and the AlpACS system is the different effect of cargo loaded adapters on the nucleotide hydrolysis. While for ParM ATP hydrolysis is accelerated by *parN*-bound ParR (Jensen and Gerdes, 1997), we show here that AlpC ATPase activity is activated by unloaded adapter AlpA. This difference may account for the apparent differences in filament dynamics. Despite sequence similarities between actin, MreB, ParM, AlpC, and other bacterial actin-like proteins filamentation occurs in different ways lending support to the notion that filamentation evolved several times separately in proteins containing an actin fold (Orlova et al., 2007).

**FIGURE 6:**
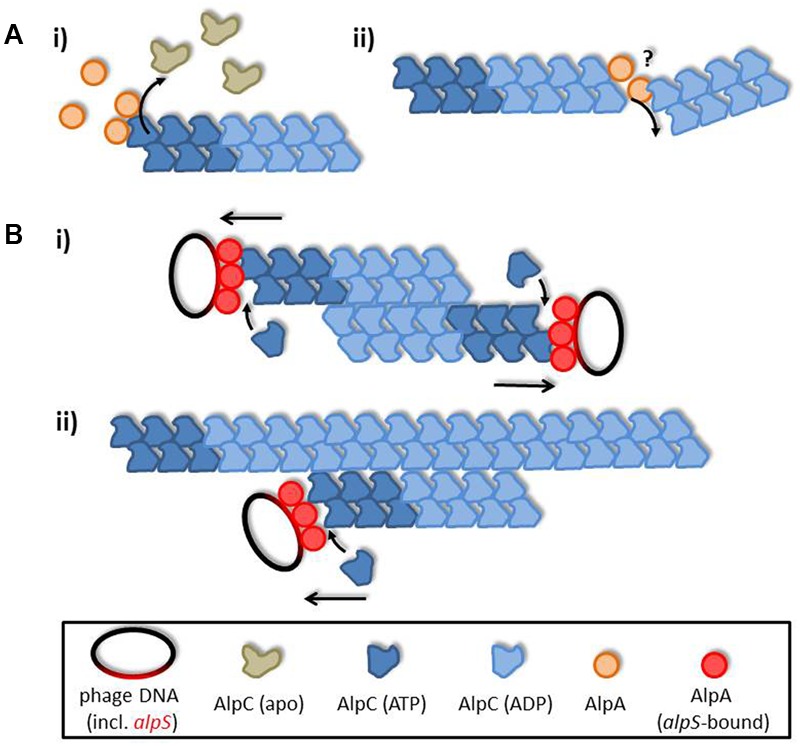
AlpA promotes AlpC filament dynamics. **(A)** Unloaded adapter AlpA (orange) induces ATP hydrolysis in AlpC (blue), thereby leading to filament disassembly. The exact mode of actin of AlpA is unclear, yet, and could either be via depolymerizing AlpC filaments from the end (i) or by levering the filament, hence, inducing filament decay (ii). **(B)** Once loaded adapter proteins (AlpA–*alpS* nucleoprotein complex) interact with AlpC filaments these are stabilized and continue to grow, thereby segregating phage DNA. Interaction of AlpC filaments could be either (i) antiparallel, or (ii) AlpC filaments could track on pre-existing AlpC structures. In summary, action of AlpA leads to a simple search and capture mechanism ensuring efficient phage DNA segregation.

Based on the observed interactions we propose a simple search and capture mechanism of viral DNA segregation by AlpAC illustrated in **Figure 6**. Upon induction of phage gene expression AlpC spontaneously nucleates and starts filamentation. Once this initial AlpC structure encounters an unloaded AlpA it rapidly disassembles, due to increased ATP turnover. Rapid depolymerization increases the pool of free AlpC that in turn will facilitate new nucleation. Furthermore, the depolymerizing function of AlpA makes other disassembly factors and a catastrophic decay mechanism unnecessary. If an AlpC filament contacts an *alpS* loaded AlpA adapter, filament formation is stabilized and continues growing, thereby segregating the viral DNA within the host cell. Negative stain data suggest a difference in filament structure in presence of absence of AlpA. It remains to be tested whether AlpC filaments in absence of AlpA–*alpS* have an altered dynamic behavior. In summary, our data suggest that the AlpAC/*alpS* segrosome works in a way similar to the well-described AlfAB/*parN* plasmid segregations system. Although the adapter proteins AlpA and AlfB are not homologous, but evolutionary pressure has led to similar segregation systems.

## Author Contributions

AF, CD, and MB conceived the study and wrote the manuscript. AF, NA, AK, and CD performed the experiments and constructed the strains. AF, AK, CD, and MB analyzed the data. MB supervised the project and provided financial support. AF, CD, and MB edited the manuscript. All authors read and approved the final article.

## Conflict of Interest Statement

The authors declare that the research was conducted in the absence of any commercial or financial relationships that could be construed as a potential conflict of interest.
