# Supplementary material for: Polymerization Dynamics of the Prophage-Encoded Actin-Like Protein AlpC Is Influenced by the DNA-Binding Adapter AlpA
Source: Front Microbiol. 2017 Aug 2;8:1429. doi: 10.3389/fmicb.2017.01429 (PMC5539076; doi:10.3389/fmicb.2017.01429)
Supplement: Supplementary file 1 [file Data_Sheet_1.DOCX]

**Supplemental Material**

# Polymerization dynamics of the prophage-encoded actin-like protein AlpC is influenced by the DNA-binding adapter AlpA

Aaron J. Forde*, Nadine Albrecht, Andreas Klingl, Catriona Donovan, & Marc Bramkamp^1^

Ludwig-Maximilians-Universität München, Fakultät für Biologie, Großhaderner Str. 2-4, 82152 Planegg-Martinsried, Germany.

* Present address: University Freiburg, Medical Center, Center for Chronic Immunodeficiency, Breisacher Str. 115, 79106 Freiburg, Germany

^1^ Corresponding author:

Marc Bramkamp

Email: marc.bramkamp@lmu.de; Phone: +49-89-218074611; Fax: +49-89-218074621

**Supplemental Material Table S1. Bacterial strains used in this study.**

| **Strain (*E. coli*)** | **Genotype** | **Reference** |
| --- | --- | --- |
| DH5α | F– Φ80*lac*ZΔM15 Δ(*lac*ZYA-*arg*F) U169 *rec*A1 *end*A1 *hsd*R17 (rK–, mK+) *pho*A *sup*E44 λ– *thi*-1 *gyr*A96 | Invitrogen |
| Lemo21(DE3) | *huA2 [lon] ompT gal (λ DE3) [dcm] ∆hsdS/ pLemo*(Cam^R^)  *λ DE3 = λ sBamHIo ∆EcoRI-B int::(lacI::PlacUV5::T7 gene1) i21 ∆nin5* pLemo *=* pACYC184*-PrhaBAD-lysY* | New England Biolabs |

**Supplemental Material Table S2. Plasmids used in this study.**

| **Plasmid** | **Resistance** | **Reference** |
| --- | --- | --- |
| pET16B-*GFP-AlpC* | Carbenicillin | (Donovan *et al.*, 2015) |
| pET16B-*mCH-AlpA* | Carbenicillin | This study |
| pET16B-*AlpC*  (pCD115) | Carbenicillin | (Donovan *et al.*, 2015) |
| pET16B-*AlpC-D301A*  (pCD116) | Carbenicillin | (Donovan *et al.*, 2015) |
| pET-TEV-*AlpA* | Kanamycin | (Donovan *et al.*, 2015) |

**Supplemental Material Table S3. Oligonucleotides used in this study.**

| **Primer** | **Sequence** | **Tm** |
| --- | --- | --- |
| AlpA-SalI-F | CAT *GTCGAC* ATGGCTCAAAAACAGGAC | 74°C |
| AlpA-BamHI-R | CAT *GGATCC* CTAGCGACCGAACGCTTG | 79°C |
| mCherry-Xho1  F | CAT *CTCGAG* ATGGTGAGCAAGGG | 69°C |
| mCherry-Sal1 R | CAT *GTCGAC* CTTGTACAGCTCGT | 67°C |
| alpS Forward | C TTAATTGTC *CTCGAG* TTAATTGTCACTTCGTTAATTGTCACTTCGTTAATTGTCACTTCGTTAATTGTCACTTCG  TTAATTGTCACTTCGTTAATTGTCCATGTC A | 78°C |
| alpS Reverse | *A* GACATGGA  CAATTAACGAAGTGACAATTAACGAAGTGACAATTAACGAAGTGACAATTAACGAA GTGACAATTAACGAAGTGACAATTAA*CTCGAG*GACAATTAA GCATG | 78°C |
| alps Scrambled  Forward | *C* GTATTATGT *CTCGAG* GTATTATGT ACTTCG GTATTATGT ACTTCG GTATTATGT ACTTCG GTATTATGT ACTTCG GTATTATGT ACTTCG GTATTATGT A | 76°C |
| alpS Scrambled  Reverse | *A* CATAATACCGAAGTACATAATACCGAAGTACATAATACCGAAGTACATAATACCGAA GTACATAATACCGAAGTACATAATAC*CTCGAG*ACATAATAC GCATG | 75°C |

**

**

**Figure S1: Purification of recombinant GFP-AlpC and mCherry-AlpA.** (**A**) GFP-AlpC and mCherry-AlpA were purified via metal affinity chromatography. Shown are elution profiles above images of Coomassie blue stained SDS-PAGE gels. (**B**) Elution profiles from size exclusion chromatography. GFP-AlpC was run on a Superdex 200 column and mCherry-AlpA on a Superose 6 column. Protein from peak fractions were separated on SDS-PAGE gels stained with Coomassie blue. Note the apparent reduction in degradation products during the second chromatography step. Molecular mass standard is indicated in kDa.


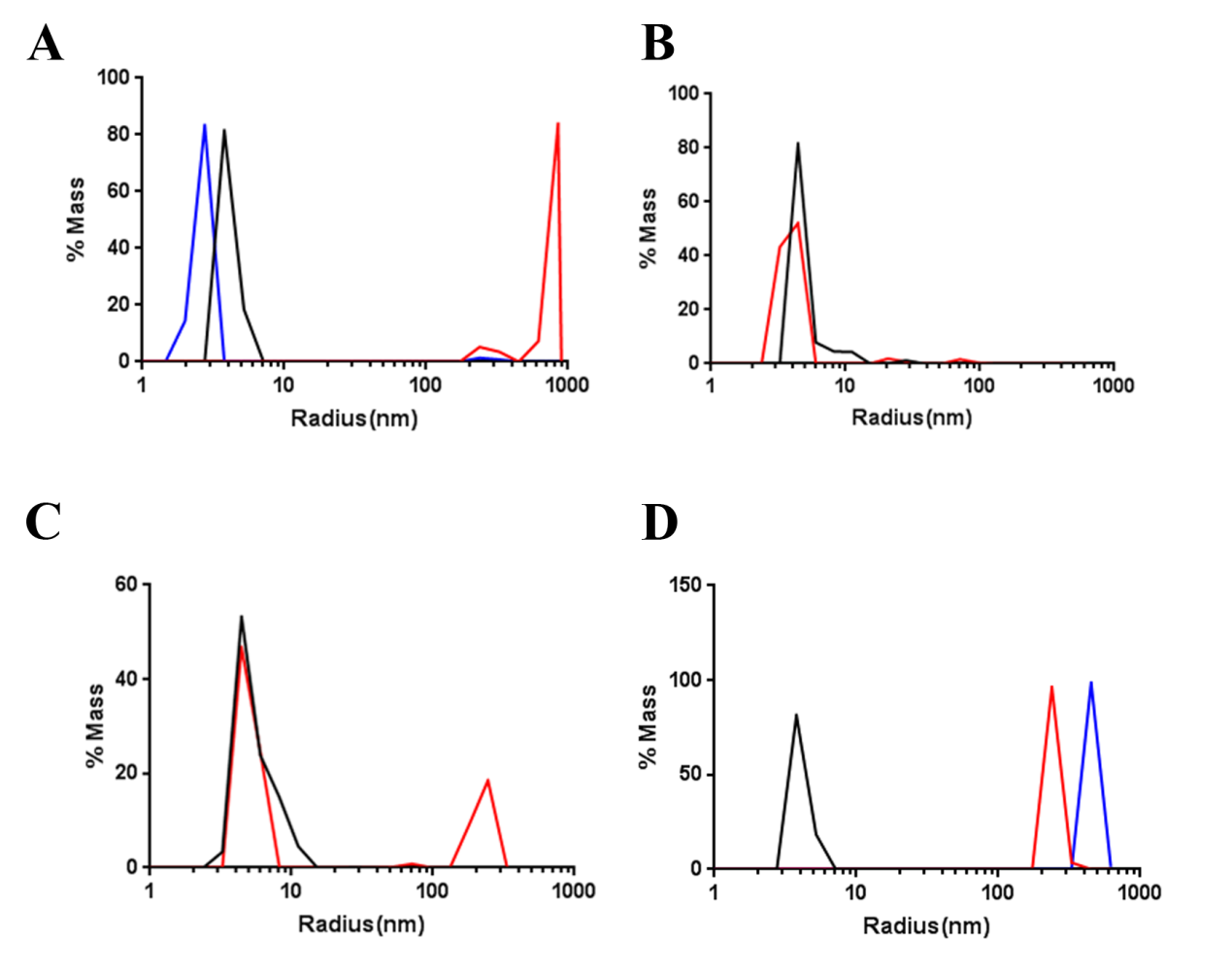


**Figure S2. Polymerization behavior of native AlpC**. To control for any effect of the fluorescent fusions used in the polymerization study non-tagged versions of AlpC and AlpA have been used. (**A**) The radius of AlpC (black line) without nucleotides is in the range of mono- or dimers. Upon ATP addition AlpC polymerizes into large polymers (red line). Addition of equimolar concentrations of AlpA leads to AlpC polymer disassembly even in presence of ATP (blue line) (**B**) A catalytically inactive AlpC mutant (AlpC-D301A) does not form polymers upon ATP addition. (**C**) Addition of ADP to AlpC promotes polymerization (red line). Note that a majority of AlpC is still found in the mono/dimeric fraction when incubated with ADP. (**D**) When AlpA was preincubated with *alpS* site containing plasmids the AlpC polymers were stabilized (blue curve).


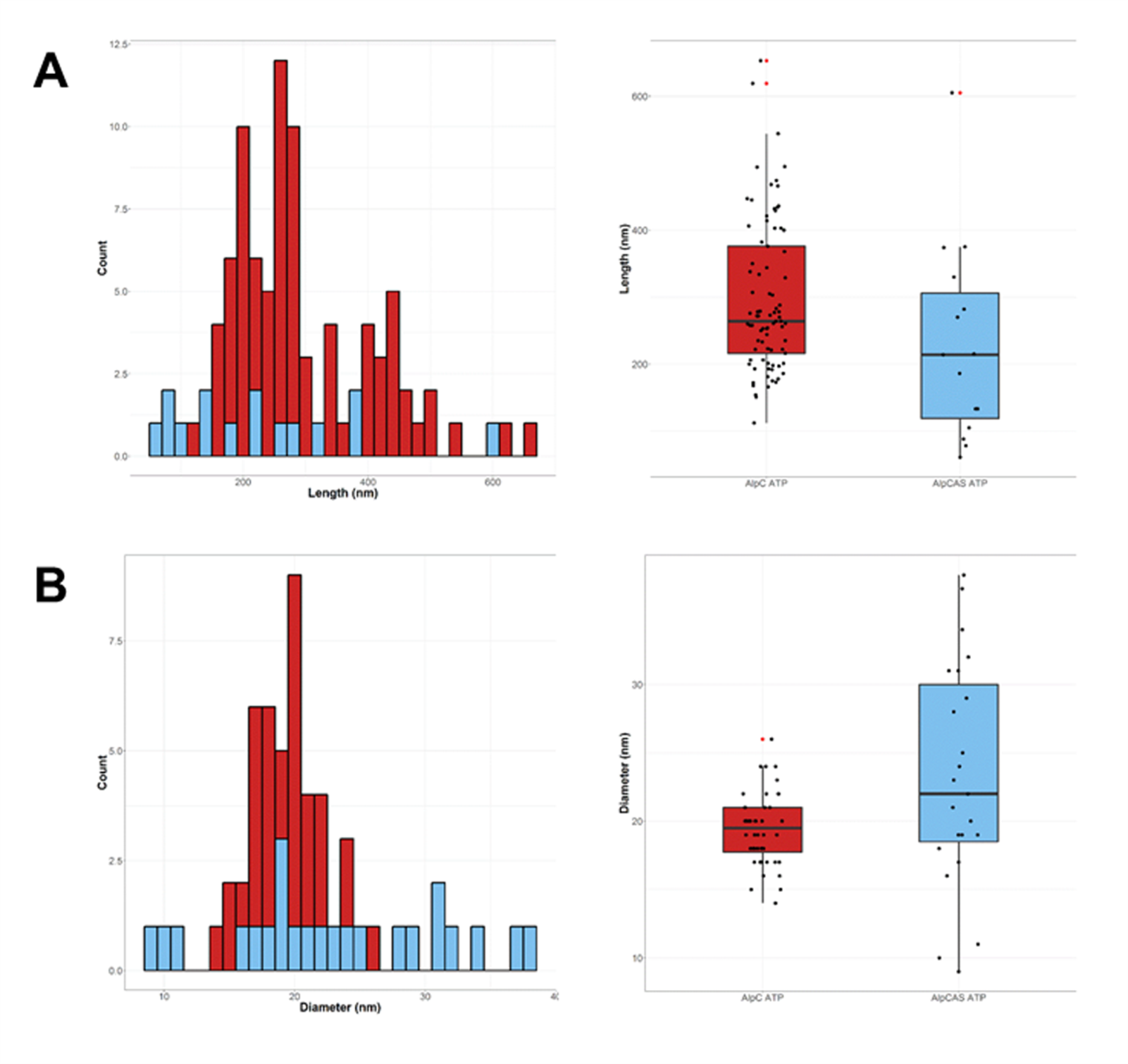


**Figure S3. Statistical analysis of AlpC filament formation analyzed by negative stain electron microscopy**. (**A**) Shown in red are filament length measured for AlpC plus ATP and shown in blue are data derived from filaments that formed in presence of AlpC, AlpA and *alpS* (in presence of ATP). (**B**) Diameter of AlpC filaments with color coding as above.
